# Impact of olfactory function on the trajectory of cognition, motor function, and quality of life in Parkinson’s disease

**DOI:** 10.3389/fnagi.2024.1329551

**Published:** 2024-03-04

**Authors:** Chia-Yen Lin, Yu-Shan Tsai, Ming-Hong Chang

**Affiliations:** ^1^Department of Neurology, Neurological Institute, Taichung Veterans General Hospital, Taichung, Taiwan; ^2^Department of Post-Baccalaureate Medicine and Brain and Neuroscience Research Center, College of Medicine, National Chung Hsing University, Taichung, Taiwan

**Keywords:** olfactory dysfunction, dysosmia, cognition, motor, quality of life, Parkinson’s disease

## Abstract

**Background:**

Olfactory dysfunction in Parkinson’s disease (PD) is associated with more severe phenotypes, but trajectories of cognitive function, disease severity, and subdomains of quality-of-life measurements in patients with distinct olfactory profiles remain underexplored.

**Objective:**

To analyze the influence of olfaction on trajectories of clinical parameters in patients with PD.

**Design:**

Retrospective cohort study.

**Subjects:**

From October 2016 to May 2021, the study tracked 58 participants over 3 years. Participants completed follow-up assessments using tools including the Chinese version of the University of Pennsylvania’s Smell Identification Test (UPSIT), Montreal Cognitive Assessment (MoCA), Movement Disorder Society-sponsored revision of the Unified Parkinson’s Disease Rating Scale, and the Chinese translation of the 39-item Parkinson’s Disease Questionnaire (PDQ-39).

**Methods:**

Participants were divided into anosmia (UPSIT < 19) and non-anosmia (UPSIT ≥ 19) groups based on initial scores. Generalized estimating equations and repeated measures correlations were used to examine longitudinal associations and correlations between olfaction and clinical parameters.

**Results:**

Divergent cognitive trajectories were observed between groups. The anosmia group exhibited a faster cognitive decline (adjusted B [beta coefficient] = −1.8, *p* = 0.012) according to the interaction effect of olfaction and time on the MoCA score. The anosmia group exhibited no longitudinal correlation between cognition and olfactory function but showed correlations with age (*r*_*rm*_ [coefficient of repeated measures correlation] = −0.464, *p* = 0.004) and disease duration (*r*_*rm*_ = −0.457, *p* = 0.005). The non-anosmia group’s UPSIT scores decreased over time (*B* = −2.3, *p* = 0.005) alongside a significant correlation with motor function (*r*_*rm*_ = −0.479, *p* = 0.006).

**Conclusion:**

The anosmia group’s accelerated cognitive decline correlated with age and disease duration, but not olfactory function, suggesting a poor cognitive outcome in this population despite the lack of longitudinal correlation between cognition and olfaction. The non-anosmia group exhibited progressive olfactory degradation and notable correlations between motor function and UPSIT scores, implying pathological accumulation in the olfactory structure and basal ganglia.

## Introduction

Olfactory dysfunction is both a precursor and a predictor of motor or non-motor symptoms of Parkinson’s disease (PD) (Fullard et al., 2017). According to Braak et al. (2003), Lewy pathology emerges in the olfactory structures and vagus nerve. Additionally, α-synuclein propagates to the neural network through prion-like behavior from the olfactory bulb (Braak et al., 2003). However, not all patients with PD exhibit features consistent with the Braak staging (Jellinger, 2019), which has led to the conceptualization of the body-first and brain-first model. This model suggests a more pronounced cognitive impairment, symmetric parkinsonism, and frequent olfactory dysfunction in the body-first category (Horsager et al., 2020, 2022).

Based on this framework, it is plausible that olfactory dysfunction correlates with worsened cognition and a decline in quality of life. Although research has attempted to unravel the relationships between olfactory dysfunction and various motor and cognitive aspects of PD, the findings have been inconclusive. Thus, there is a gap in the understanding of the trajectory of olfactory function and its correlation with motor function and cognition (Herting et al., 2008; Meusel et al., 2010; Campabadal et al., 2017; Lewis et al., 2020). While some studies have not reported an association (Doty et al., 1988; Herting et al., 2008), others have shown that baseline olfactory dysfunction in PD is associated with motor (He et al., 2020) and cognitive progression (Fullard et al., 2016; Domellöf et al., 2017; He et al., 2020) and higher use of dopaminergic medications (Lee et al., 2015; He et al., 2020).

Regarding the trajectory of olfactory function, some studies have noted relative stability over time (Doty et al., 1988). However, others have reported non-linear deterioration (Herting et al., 2008) and significant changes particularly in individuals with better initial olfactory performance (Meusel et al., 2010; Domellöf et al., 2017). Our previous cross-sectional study observed a correlation between anosmia and worse quality of life (QoL). This was manifested in activities of daily living (ADL) and cognition (COG) according to assessments by the Chinese translation of the 39-item Parkinson’s Disease Questionnaire (PDQ-39). We recognized the need for deeper insight into the roles of olfactory function in shaping QoL trajectories in PD patients (Lin et al., 2022).

The literature has acknowledged that there is a progressive decline across different disease stages concerning olfactory function and QoL. Nevertheless, the influence of olfactory function on QoL subdomains remains an understudied area (Lewis et al., 2020). To address this gap, this study examined the impact of olfactory function on the trajectories of cognitive function, motor function, and QoL in PD.

## 2 Materials and methods

### 2.1 Study design

We initiated this study at Taichung Veterans General Hospital, recruiting participants from October 2016 to May 2021. The inclusion criterion was adherence to the International Parkinson and Movement Disorder Society Clinical Diagnostic Criteria for Parkinson’s disease (Postuma et al., 2015). The visiting periods were scheduled as follows: visit 1 (0–12 months), visit 2 (13–24 months), visit 3 (25–36 months), and so on, post the initial olfactory evaluation. We recorded essential demographic data and utilized various validated instruments for the assessments. To be eligible for analysis, participants had to complete the assessments described below during their first visit: the traditional Chinese version of University of Pennsylvania Smell Identification Test (UPSIT) (Doty et al., 1984; Jiang et al., 2010; Yu and Wu, 2014), the Montreal Cognitive Assessment (MoCA) (Nasreddine et al., 2005), the Movement Disorder Society-sponsored revision of the Unified Parkinson’s Disease Rating Scale (M-UPDRS) (Goetz et al., 2008), and the Chinese-translated version of the 39-item Parkinson’s Disease Questionnaire (PDQ-39) (Peto et al., 1995; Ma et al., 2005). We tracked these measures longitudinally during each visit.

We excluded visits without UPSIT evaluation or with errors/inapplicable data in any test component. Only the most comprehensive and latest data from the initial evaluation were retained, discarding any other evaluations within the same period. Finally, we excluded those who underwent deep brain stimulation surgery during the follow-up period (Supplementary Figure 1 for flow diagram of inclusion of this study). This study was approved by the Institutional Review Board and Ethics Committee of Taichung Veterans General Hospital (No. CE22189B). Participants provided written informed consent prior to enrollment, in accordance with the ethical standards addressed in the Declaration of Helsinki.

### 2.2 Parameters of olfactory function, cognitive function, disease severity, and quality of life (QoL)

We evaluated each participant’s olfaction using the traditional Chinese version of the University of Pennsylvania’s Smell Identification Test (UPSIT) (Sensonics, Inc., Haddon Heights, NJ, USA). The subjects received one point for correctly identifying the odorant out of four in a sample of forty items. This method had previously been validated (Doty, 2019). A previously reported threshold was used to distinguish total anosmia (UPSIT < 19) from other olfactory statuses across various age groups, as this threshold aligns closely with the average UPSIT values (17–21) seen in PD patients (Picillo et al., 2014; Yu and Wu, 2014; Doty, 2019). Using this approach, patients with normosmia and mild to severe microsmia were grouped into the non-anosmia category (UPSIT ≥ 19). This categorization based on olfactory status has also been utilized in other relevant literature (Iranzo et al., 2021). We categorized patients based on their baseline UPSIT scores. The UPSIT scores were tracked at each visit.

In this study, we utilized the Montreal Cognitive Assessment (MoCA) to evaluate global cognitive function. To quantify disease severity, we employed the Movement Disorder Society-sponsored revision of the Unified Parkinson’s Disease Rating Scale (M-UPDRS), reporting in the “On” state to mitigate the carryover effects of dopaminergic medications. We focused on the total score and the Part III score to gauge overall disease severity and objective motor function, respectively.

We employed the Chinese-translated version of the 39-item Parkinson’s Disease Questionnaire PDQ-39 to evaluate quality of life (QoL) in patients with PD, a reliable and validated tool acknowledged for its efficiency in evaluating QoL in PD patients (Peto et al., 1995; Ma et al., 2005; Martinez-Martin et al., 2011). The PDQ-39 encompasses eight dimensions, including: mobility (10 items), activities of daily living (ADL, six items), emotional wellbeing (six items), stigma (four items), social support (three items), cognition (COG, four items), communication (three items), and bodily discomfort (three items). Given our preceding research, which evidenced a correlation between anosmia and both ADL and COG in cross-sectional studies (Lin et al., 2022), we concentrated our analysis on the summary index (SI) alongside the ADL and COG dimensions. Each dimension possesses a score ranging between 0 and 100, with the SI representing the mean score of all domains.

### 2.3 Statistical analysis

We carried out statistical analysis using SPSS version 22.0 for Windows (SPSS Inc., Chicago, IL, USA). The normal distribution of demographic data and scores from various tests (UPSIT, MoCA, M-UPDRS, and PDQ-39) was verified using the Kolmogorov-Smirnov test. During the initial visit, we applied the Mann-Whitney *U*-test and Fisher’s exact test to compare continuous and categorical variables, respectively, between the total anosmia group (UPSIT < 19) and the non-anosmia group (UPSIT ≥ 19).

To examine the effect of time on different measures (including LEDD, UPSIT, MoCA, M-UPDRS, and PDQ-39 scores) throughout the follow-up, we used a generalized estimating equation (GEE) model with an exchangeable covariate structure. This approach accommodated the uneven follow-up intervals and differences between the groups at the initial visit.

We employed another GEE model to explore the potential impact of various degrees of olfactory function on the progression rates of disease severity and quality of life (QoL). In this model, olfactory function was categorized based on UPSIT scores either <19 (anosmia) or ≥19 (non-anosmia) at initial visit. The visit timeline was used as a covariate. We evaluated the interaction between olfaction and time, allowing us to pinpoint the influences of olfaction, time, and their interrelation (olfaction × time) on the parameters under investigation.

We investigated the longitudinal impact of UPSIT scores on clinical metrics including M-UPDRS Part III and MoCA, using linear mixed-effect models (Bates et al., 2015). Adjustments for age, sex, and disease duration were made to reflect the nuanced interplay over time.

To examine the existence of a floor effect in olfactory function, we hypothesized that patients with notable olfactory deficits would not demonstrate correlational changes with other clinical measures, unlike individuals with preserved olfaction. This premise was explored using repeated measures correlation analysis (Bakdash and Marusich, 2017) in RStudio, which examined the relationship between olfactory function and clinical indicators, further categorized into anosmia and non-anosmia groups based on their olfactory capabilities.

In all analyses, we considered *p* < 0.05 in a two-tailed test to be statistically significant.

## 3 Results

Table 1 presents information on patients who attended at least two comprehensive evaluations during the first three visits, who were divided into an anosmia group and a non-anosmia group. There were 32 patients in the anosmia group and 26 patients in the non-anosmia group. In the anosmia group, 25 patients were retained at visit 2, and 10 patients were retained at visit 3. In the non-anosmia group, 19 patients were retained at visit 2, and 11 patients were retained at visit 3. The participants’ ages and scores on part III of the M-UPDRS were normally distributed, while other variables were not. Therefore, the median values and interquartile ranges are reported with *p*-values indicating statistical significance. Due the limited data from visits 4 and 5, the primary analysis pertained to the first three visits.

**TABLE 1:** Anosmia vs. non-anosmia: initial comparison based on first visit and aggregate of the three visits with ≥2 clinical assessments.

	Visit 1			Visit 2		Visit 3	
	**Anosmia *n* = 32**	**Non-anosmia *n* = 26**	***p***	**Anosmia *n* = 25**	**Non-anosmia *n* = 19**	**Anosmia *n* = 10**	**Non-anosmia *n* = 11**
Age, year	64 (61–70)	66 (58–74)	0.956	65 (60–71)	65 (59–77)	73 (65–81)	66 (60–69)
Sex, male (%)	23 (72%)	17 (65%)	0.776	18 (72%)	11 (58%)	7 (70%)	9 (82%)
Sex, female (%)	9 (28%)	9 (35%)		7 (28%)	8 (42%)	3 (30%)	2 (18%)
Duration, month	49 (27–108)	31 (12–56)	0.043*	79 (58–131)	52 (29–71)	59 (34–107)	64 (44–100)
Follow-up, month	0 (0–0)	0 (0–0)	1.000	16 (14–21)	15 (14–22)	26 (25–30)	29 (26–34)
LEDD, mg	655 (175–937)	250 (108–457)	0.009*	798 (423–1297)	400 (250–639)	724 (433–1023)	524 (400–948)
UPSIT	14 (12–16)	22 (20–25)	0.000*	12 (10–15)	21 (18–24)	13 (11–15)	21 (18–23)
MOCA	27 (21–29)	28 (24–29)	0.374	24 (20–28)	27 (23–29)	23 (14–27)	28 (27–29)
**M-UPDRS**							
Total	54 (37–72)	44 (31–57)	0.051	50 (37–82)	47 (34–53)	59 (48–64)	55 (24–63)
Part III	34 (21–42)	25 (18–35)	0.057	31 (23–42)	29 (23–36)	38 (33–43)	30 (17–33)
**PDQ-39**							
SI	19 (9–30)	19 (6–30)	0.690	14 (6–33)	14 (9–27)	18 (8–41)	19 (7–30)
ADL	13 (1–29)	6 (0–23)	0.211	13 (0–27)	4 (0–21)	15 (0–31)	4 (0–21)
COG	25 (14–44)	19 (11–39)	0.299	19 (6–47)	19 (0–38)	28 (9–56)	25 (13–56)

Data are presented as median (Q1–Q3), with “duration” denoting time since symptom onset and “follow-up” representing time from initial visit. Anosmia: baseline UPSIT < 19; Non-anosmia: baseline UPSIT ≥ 19. ADL, activities of daily living; COG, cognitions; LEDD, levodopa equivalent daily dose; MoCA, Montreal cognitive assessment; M-UPDRS, movement disorder society-sponsored revision of the Unified Parkinson’s Disease Rating Scale; PDQ-39, Chinese-translated version of 39-item Parkinson’s Disease Questionnaire; SI, summary index; UPSIT, traditional Chinese version of the University of Pennsylvania Smell Identification Test.

*p < 0.05.

Initially, the median UPSIT scores representing olfactory function were 14 for the anosmia group and 22 for the non-anosmia group. The anosmia group was associated with longer disease duration (49 vs. 31 months, *p* = 0.017) and a higher Levodopa equivalent daily dose (LEDD) (655 vs. 250 mg, *p* = 0.007). Although the anosmia group had higher total scores (54 vs. 44, *p* = 0.051) and part-III scores (34 vs. 25, *p* = 0.057) on the M-UPDRS, these differences were not statistically significant. Both groups exhibited comparable QoL according to the similar PDQ-39 SI, ADL, and COG values.

**FIGURE 1:**
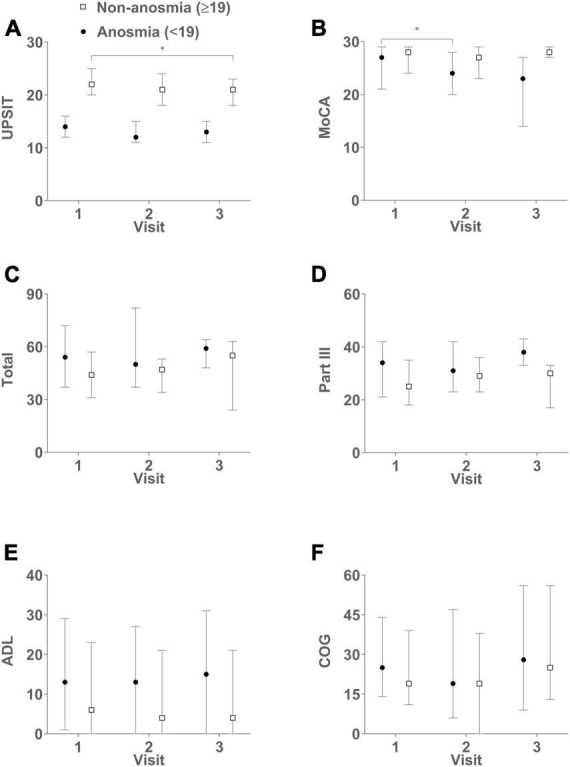
Complements Tables 1, 2, illustrating the dynamic changes in clinical assessments over successive visits. The figures in the six panels represent median scores with interquartile range (IQR) for both groups: UPSIT **(A)**, MoCA **(B)**, Total **(C)**, Part III **(D)**, ADL **(E)**, and COG **(F)**. Higher UPSIT and MoCA scores indicate better olfactory and cognitive functions, respectively. Conversely, higher scores in total, part III, ADL, and COG signify greater disease severity and reduced QoL. The non-anosmia group exhibits a significant decline in UPSIT scores when comparing visit 3 to visit 1 (*B* = –2.3, *p* = 0.005). Meanwhile, the anosmia group displays a decrease in MoCA scores when comparing visit 2 to visit 1 (*B* = –2.8, *p* = 0.005). Anosmia: baseline UPSIT < 19; Non-anosmia: baseline UPSIT ≥ 19. *p < 0.05.

Table 2 and Figure 1 illustrate the trajectory of clinically assessments according to the generalized estimating equation (GEE) model. A decline in the UPSIT score was observed in the non-anosmia group between visits 3 and 1 (*B* [beta coefficient] = −2.3, *p* = 0.005), and a decline in the MoCA score was seen between the first two visits in the anosmia group (*B* = −2.8, *p* = 0.005). Both groups exhibited significant increases in LEDD. However, other health indicators remained relatively stable over the visits. As shown in Table 3, further GEE analysis indicated interactions between time and olfaction. A more pronounced cognitive decline occurred over time in the anosmia group, and this trend persisted even after adjusting for demographic variables and LEDD (crude *B* = −1.8, *p* = 0.013; adjusted *B* = −1.8, *p* = 0.012).

**TABLE 2:** Clinical assessment trajectories in anosmia and non-anosmia groups over visits: GEE analysis.

	Anosmia				Non-anosmia			
	**Visit 2 vs. visit 1**		**Visit 3 vs. visit 1**		**Visit 2 vs. visit 1**		**Visit 3 vs. visit 1**	
	**Crude**		**Crude**		**Crude**		**Crude**	
	***B***	***P***	***B***	***p***	***B***	***p***	***B***	***p***
LEDD	204	<0.001*	253	0.004*	141	<0.001*	352	0.003*
UPSIT	−1.1	0.165	−0.1	0.893	−1.2	0.267	−2.3	0.005*
MoCA	−2.8	0.005*	−3.0	0.052	−0.7	0.274	0.2	0.831
**M-UPDRS**								
Total	−2.1	0.700	1.8	0.653	−0.1	0.960	4.0	0.430
Part III	−1.2	0.638	2.9	0.261	2.0	0.258	3.2	0.283
**PDQ-39**								
SI	−4.7	0.095	3.6	0.257	−1.5	0.673	−0.4	0.883
ADL	−6.0	0.157	1.8	0.594	−3.3	0.340	−4.1	0.274
COG	−5.4	0.186	2.1	0.725	−6.0	0.304	5.8	0.306

Results indicate a decrease in the UPSIT scores from baseline in the non-anosmia group (*B* = −2.3, *p* = 0.005) and a decline in the MoCA scores from baseline in the anosmia group (*B* = −2.8, *p* = 0.005). Anosmia: baseline UPSIT < 19; Non-anosmia: baseline UPSIT ≥ 19. Table 1 and the above table are related to Figure 1. ADL, activities of daily living; *B*, beta coefficient; COG, cognitions; GEE, generalized estimating equation; LEDD, levodopa equivalent daily dose; MoCA, Montreal cognitive assessment; M-UPDRS, Movement Disorder Society-sponsored revision of the Unified Parkinson’s Disease Rating Scale; PDQ-39, Chinese-translated version of 39-item Parkinson’s Disease Questionnaire; SI, summary index; UPSIT, traditional Chinese version of the University of Pennsylvania Smell Identification Test.

*p < 0.05.

**TABLE 3:** Olfaction and time interaction effects on clinical assessment trajectories: GEE analysis.

	Crude model				Adjusted model			
	**Olfaction**		**Olfaction × Time**		**Olfaction**		**Olfaction × Time**	
	**Crude**		**Crude**		**Adjusted**		**Adjusted**	
	***B***	***p***	***B***	***p***	***B***	***p***	***B***	***P***
Age	0.2	0.950	0.1	0.191				
Duration	28.7	0.035*	−0.4	0.644				
LEDD	326.4	0.006*	−16.5	0.785				
UPSIT	−10.3	<0.001*	0.8	0.239	−10.0	<0.001*	0.9	0.176
MOCA	0.6	0.651	−1.8	0.013*	1.3	0.297	−1.8	0.012*
**M-UPDRS**								
Total	15.3	0.056	−1.5	0.659	10.9	0.179	−1.9	0.602
Part III	7.9	0.080	−1.0	0.603	7.7	0.080	−1.3	0.517
**PDQ-39**								
SI	1.9	0.735	0.0	0.998	−2.0	0.707	0.0	0.985
ADL	3.7	0.615	0.9	0.771	−2.4	0.755	1.2	0.722
COG	6.5	0.405	−2.1	0.579	1.1	0.887	−2.3	0.565

The olfaction effects were compared between anosmia and non-anosmia groups. Adjustments were made for age, sex, disease duration, and LEDD. Data indicates a faster cognitive decline in the anosmia group (olfaction** ×** time effect on MoCA scores adjusted *B* = −1.8, *p* = 0.012). Anosmia: baseline UPSIT < 19; Non-anosmia: baseline UPSIT ≥ 19. ADL, activities of daily living; *B*, beta coefficient; COG, cognitions; GEE, generalized estimating equation; LEDD, levodopa equivalent daily dose; MoCA, Montreal cognitive assessment; M-UPDRS, Movement Disorder Society-sponsored revision of the Unified Parkinson’s Disease Rating Scale; PDQ-39, Chinese-translated version of 39-item Parkinson’s Disease Questionnaire; SI, summary index; UPSIT, traditional Chinese version of the University of Pennsylvania Smell Identification Test.

*p < 0.05.

**FIGURE 2:**
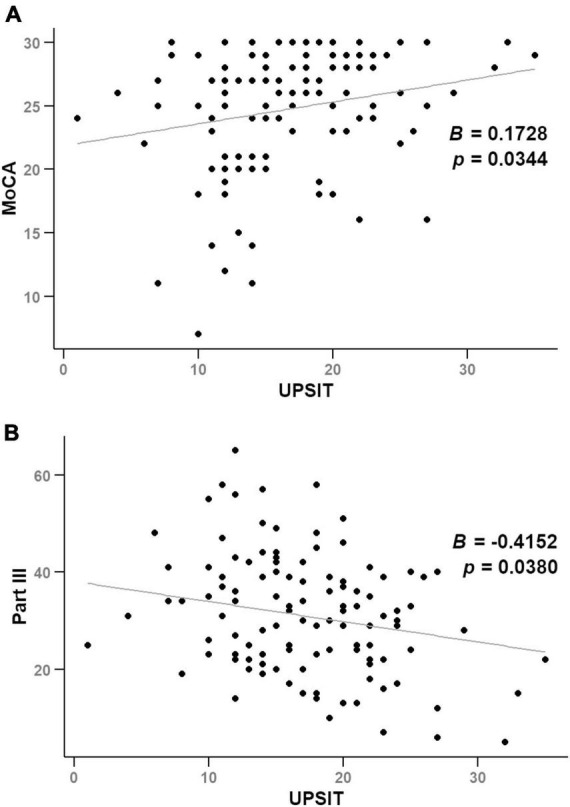
Illustrates scatter plots of UPSIT scores against MoCA **(A)** and Part III scores **(B)** over time, analyzed using linear mixed-effects models.

Figure 2 and Supplementary Table 1 demonstrate significant correlations between UPSIT score and MoCA score (*B* = 0.1728, *p* = 0.0344) and Part III score (*B* = −0.4152, *p* = 0.0380) over time, after adjusting for age, sex, and disease duration in a cohort of 58 participants.

**FIGURE 3:**
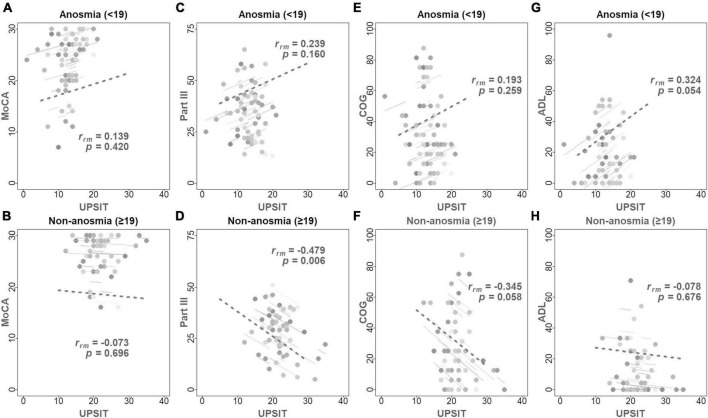
Displays repeated measures correlation coefficients (*r*_*rm*_) with the fitted *r*_*rm*_ represented by parallel solid lines, complementing Table 4. Higher UPSIT and MoCA scores represent enhanced olfactory and cognitive functions, respectively, while increased scores in part III, ADL, and COG denote advanced disease severity and decreased QoL. The top row of panels illustrates the correlations between UPSIT scores and MoCA **(A)**, Part III **(C)**, COG **(E)**, and ADL **(G)** scores in the anosmia group. The bottom row **(B,D,F,H)** depicts these correlations for the non-anosmia group. Significantly, panel **(D)** reveals a negative correlation between UPSIT scores and motor function in the non-anosmia group (*r*_*rm*_ = –0.479, *p* = 0.006). Anosmia: baseline UPSIT < 19; Non-anosmia: baseline UPSIT ≥ 19.

Table 4 and Figure 3 show the longitudinal correlations between the clinical assessments and different variables, including demographic factors and UPSIT scores. Notably, the anosmia group exhibited a negative correlation between cognitive function and both age (*r*_*rm*_ [coefficient of repeated measures correlation] = −0.464, *p* = 0.004) and disease duration (*r*_*rm*_ = −0.457, *p* = 0.005), but not with olfactory function. Conversely, the non-anosmia group revealed a strong linkage between deteriorating motor function and age (*r*_*rm*_ = 0.418, *p* = 0.019), disease duration (*r*_*rm*_ = 0.360, *p* = 0.047), and reduced olfactory function (*r*_*rm*_ = −0.479, *p* = 0.006). This indicated that impaired motor function was correlated with older age, longer disease duration, and worse olfactory function in the non-anosmia group.

**TABLE 4:** Longitudinal correlation of clinical assessments with demographic factors and UPSIT: Rmcorr analysis.

	MoCA		Part III		COG		ADL	
	***r*_*rm*_**	***p***	***r*_*rm*_**	***p***	***r*_*rm*_**	***p***	***r*_*rm*_**	***p***
**Anosmia**								
Age	−0.464	0.004*	0.156	0.364	−0.082	0.634	−0.039	0.822
Duration	−0.457	0.005*	0.071	0.681	−0.099	0.565	−0.104	0.546
LEDD	−0.213	0.213	−0.192	0.262	−0.238	0.162	−0.438	0.007*
UPSIT	0.139	0.420	0.239	0.160	0.193	0.259	0.324	0.054
**Non-anosmia**								
Age	−0.166	0.373	0.418	0.019*	0.074	0.694	−0.081	0.665
Duration	−0.118	0.528	0.360	0.047*	0.081	0.665	−0.147	0.432
LEDD	−0.030	0.872	0.120	0.520	−0.011	0.951	−0.242	0.190
UPSIT	−0.073	0.696	−0.479	0.006*	−0.345	0.058	−0.078	0.676

The above table shows a negative correlation between UPSIT scores and part III scores in the non-anosmia group (*r*_*rm*_ = −0.479, *p* = 0.006), related to Figure 3. Anosmia: baseline UPSIT < 19; Non-anosmia: baseline UPSIT ≥ 19. ADL, activities of daily living of PDQ-39; COG, cognitions of PDQ-39; LEDD, levodopa equivalent daily dose; MoCA, Montreal cognitive assessment; Part III, part III of movement disorder society-sponsored revision of the Unified Parkinson’s Disease Rating Scale; PDQ-39, Chinese-translated version of 39-item Parkinson’s Disease Questionnaire; Rmcorr, repeated measures correlation; *r*_*rm*_, coefficient or repeat measurement correlation; UPSIT, traditional Chinese version of the University of Pennsylvania Smell Identification Test.

*p < 0.05.

Additionally, the non-anosmia group showed a marginal negative correlation between PDQ-39 COG and UPSIT scores (*r*_*rm*_ = −0.345, *p* = 0.058), hinting at a trend where worsened olfactory function parallels decreases in cognitive aspects of QoL. Conversely, the anosmia group exhibited better ADL scores correlating with higher LEDD (*r*_*rm*_ = −0.438, *p* = 0.007) and slightly worse UPSIT scores (*r*_*rm*_ = 0.324, *p* = 0.054). This indicates a somewhat unexpected relationship between olfactory function and ADL.

### 3.1 Sensitivity analysis

We performed a sensitivity analysis of patients who completed two comprehensive evaluations during the first two visits. Details are shown in Supplementary Tables 2–5, which indicate a consistent trend. Supplementary Table 3 shows that there was a cognitive decline in the anosmia group, which was underscored by the significantly different MoCA scores between the first and second visits and is supported by the Wilcoxon signed rank test (Hodges-Lehmann median difference = −2.5, *p* = 0.003). Supplementary Table 4 illustrates that there were diverging trajectories in cognitive function between the two groups, which suggested greater deterioration of cognition in the anosmia group. This is highlighted by the interaction effect of olfaction and time on the MoCA score (crude *B* = −2.4, *p* = 0.051; adjusted *B* = −2.3, *p* = 0.062). As shown in Supplementary Table 5, the non-anosmia group exhibited a marginal negative correlation between motor function and olfactory function (*r*_*rm*_ = −0.441, *p* = 0.051).

## 4 Discussion

This 3-year study differentiated between anosmia and non-anosmia groups based on their initial UPSIT scores to understand the trajectories of olfactory function and their links to cognition, disease severity, and QoL. The baseline data highlighted that the anosmia group had a longer disease duration and greater severity. This group also had greater cognitive declines than the non-anosmia group according to the MoCA scores. Interestingly, only the non-anosmia group demonstrated a longitudinal correlation between olfactory and motor functionalities.

### 4.1 Review of the literature

Previous research has established cross-sectional correlations between olfactory dysfunction and motor disability, apathy, cognitive impairment, executive dysfunction, probable rapid eye movement sleep behavior disorder (RBD), and excessive daytime sleepiness (EDS). Notably, decreased acetylcholine levels in the CSF have been associated with both olfactory dysfunction and RBD. Executive function, closely linked to olfactory function, may also indicate compromised cholinergic neuronal transmission (Masala et al., 2018; Solla et al., 2023; Wang et al., 2023).

Longitudinal analyses present a mixed picture. Some studies reported increases in UPDRS total/II and PDQ-39 scores, a decrease in UPSIT scores, and stable MoCA and Part III scores over 3 years (Lewis et al., 2020). Others focused on baseline olfactory function, tracking changes in MMSE and UPDRS over 2 years (He et al., 2020), or attempted to find longitudinal correlations between olfactory function, UPDRS III, H&Y scores (Herting et al., 2008), and MMSE changes (Fujio et al., 2020) without significant findings. Research examined multiple olfactory assessments and the incidence of dementia, revealing no significant longitudinal correlation between olfactory function and UPDRS scores (Domellöf et al., 2017). Another study explored the relationship between changes in olfactory function and brain volume (Campabadal et al., 2017).

These findings illustrate the complex interactions between olfactory dysfunction and PD’s motor and cognitive aspects. Our study contributes to this field by providing a detailed longitudinal analysis of olfactory function, motor symptoms, cognitive performance, and quality of life, enhancing our understanding of their interrelations in PD.

### 4.2 Trajectories of olfactory function between groups

During the follow-up, the non-anosmia group showed a slight decrease in UPSIT scores. A systematic review of seven studies indicated a 14% decrease from baseline over an average of 38 months (Ercoli et al., 2022). When this rate of progression was applied to our cohort (median UPSIT of 22 vs. 14 in the anosmia vs. non-anosmia group), it indicated that there would have been a three-point decrease in UPSIT from 22 points in the non-anosmia group. This is slightly greater than the actual decline observed in our study, where the UPSIT score decreased from a median of 22 (interquartile range: 20–25) to 21 (18–23) in the non-anosmia group over a median of 29 (26–34) months. However, this difference might be minor and could have been influenced by participant dropouts.

Previous studies suggest that olfactory function tends to remain stable during motor progression, and this stability is attributed to a “floor effect.” This effect means that minor changes do not significantly impact the results of olfactory tests due to early pathological changes (Fullard et al., 2017). Although our study did not establish a definite conclusion regarding the decline in olfactory function, we did find a correlation between motor and olfactory functions in the group with preserved olfaction. This finding implies that the “floor effect” may not be a universal phenomenon in patients with PD.

### 4.3 Cognitive function trajectory and its correlation with olfactory function

We observed a pronounced decline in cognitive performance in the anosmia group according to MoCA evaluations. The two groups were similar in cognition at baseline, but the anosmia group showed a faster decline in cognition. This is in line with several studies (Fullard et al., 2016; Domellöf et al., 2017; He et al., 2020). We also investigated whether the decline in cognition correlated with olfactory function over time. There was no correlation between cognition and olfactory function, but it did have correlations with age and disease duration in the anosmia group. This observation suggests that the accelerated cognitive decline in individuals with impaired olfaction could potentially be attributed to pathologies related to aging, such as amyloid beta, tau, or cholinergic dysfunction, which is possibly exacerbated by underlying Lewy pathology (Tsuboi et al., 2003; Mundiñano et al., 2011; Fullard et al., 2016; Pasquini et al., 2021; Borghammer et al., 2022).

### 4.4 QoL trajectories and correlation with olfactory function

Our assessment of QoL over time focusing on SI, ADL, and COG revealed no discernible deterioration or variance between the two groups. Although a weak correlation between UPSIT and certain QoL parameters was noted in a previous cross-sectional study (Lin et al., 2022), this longitudinal investigation did not substantiate the finding. This difference could possibly be due to a limited sample size and considerable variations in individual data points.

### 4.5 Motor function trajectory and its correlation with olfactory function

Despite prior research indicating a significant decrease in motor functionality in the group with poor olfaction (He et al., 2020), our results contradicted this finding. This discrepancy might arise from the small sample size in our study. However, a notable finding was the correlation between olfactory and motor functions in the non-anosmia group. This relationship could potentially be explained by the steady spread of Lewy bodies from olfactory structures and supports the brain-first theory of PD, where alpha-synuclein pathology originates from the brain and propagates in a prion-like fashion in the neural network (Horsager et al., 2022).

### 4.6 Strengths and limitations

This study is distinct in terms of its longitudinal analysis of both anosmia and non-anosmia groups over a period of up to 3 years, as well as tracking the relationships between UPSIT scores and key clinical parameters such as cognitive and motor functions, QoL, and its subscales. To the best of our knowledge, this is the first report to investigate the longitudinal correlations involving UPSIT scores with motor function, cognition, and QoL. Categorizing participants based on a single cut-off point is recognized as a limitation, given the influence of age and sex on olfactory function (Brumm et al., 2023). Nevertheless, a UPSIT score of 18 is universally indicative of anosmia and is within the lower 8th and 14th percentiles for female and male populations aged over 50 years, respectively (Doty, 2019; Brumm et al., 2023).

Our study was also limited by the small sample size, which arose from our aim to monitor olfactory function, disease severity, and QoL over 3 years. The coinciding COVID-19 restrictions in Taiwan from April 2020 to December 2022, overlapping our study period (October 2016 to May 2021), significantly restricted olfactory evaluations (due to mask mandates) and impacted patient follow-up compliance. Unfortunately, the sample size limits the applicability of our findings to broader ranges of demographics, despite successfully pinpointing a discernible decline in cognitive function. Another critical limitation is the uneven follow-up periods and the incidence of missed appointments, particularly during the third series of visits, which introduced potential for attrition bias. To mitigate this, we structured the follow-up intervals as delineated in the methods section, which facilitated group comparisons over these timelines. Employing a generalized estimating equation with an exchangeable covariate structure allowed us to accommodate the variations resulting from irregular follow-up periods and missed visits in our analysis. Additionally, linear mixed-effect models facilitated handling of missing data, allowing for adjustment of covariates and evaluation of olfactory function as a continuous variable. Moreover, a sensitivity analysis was performed with patients who completed evaluations across the initial two visits, which echoed the primary trends observed in our study and affirmed the reliability of our results. Finally, our dataset currently does not include sufficient data to assess the relative risk of developing dysautonomia and RBD between anosmia and non-anosmia groups, a comparison crucial for advancing our understanding of the brain-first vs. body-first theories of PD.

## 5 Conclusion

Our result indicated a more rapid decline in cognitive function in the anosmia group than in the non-anosmia group. We didn’t find a longitudinal correlation between cognitive and olfactory functions, suggesting influences from other aging-related pathologies. However, a longitudinal correlation between olfactory and motor functions was evident in the non-anosmia group, implying a potential progression of Lewy pathology in the early olfactory system and substantia nigra.

## Data availability statement

The raw data supporting the conclusions of this article will be made available by the authors, without undue reservation.

## Ethics statement

The studies involving humans were approved by the Institutional Review Board and Ethics Committee of Taichung Veterans General Hospital (No. CE22189B). The studies were conducted in accordance with the local legislation and institutional requirements. The participants provided their written informed consent to participate in this study.

## Author contributions

C-YL: Data curation, Formal analysis, Methodology, Visualization, Writing – original draft, Writing – review and editing. Y-ST: Data curation, Investigation, Writing – review and editing. M-HC: Conceptualization, Data curation, Funding acquisition, Project administration, Resources, Supervision, Validation, Writing – original draft, Writing – review and editing.

## References

[B1] Bakdash J. Z. Marusich L. R. (2017). Repeated measures correlation. *Front. Psychol.* 8:456. 10.3389/fpsyg.2017.00456 28439244 PMC5383908

[B2] Bates D. Mächler M. Bolker B. Walker S. (2015). Fitting linear mixed-effects models using lme4. *J. Stat. Softw.* 67 1–48.

[B3] Borghammer P. Just M. K. Horsager J. Skjærbæk C. Raunio A. Kok E. H. (2022). A postmortem study suggests a revision of the dual-hit hypothesis of Parkinson’s disease. *NPJ Parkinsons Dis.* 8:166. 10.1038/s41531-022-00436-2 36450732 PMC9712280

[B4] Braak H. Rüb U. Gai W. P. Del T. K. (2003). Idiopathic Parkinson’s disease: Possible routes by which vulnerable neuronal types may be subject to neuroinvasion by an unknown pathogen. *J. Neural Transm.* 110 517–536. 10.1007/s00702-002-0808-2 12721813

[B5] Brumm M. C. Pierz K. A. Lafontant D.-E. Caspell-Garcia C. Coffey C. S. Siderowf A. (2023). Updated percentiles for the university of pennsylvania smell identification test in adults 50 years of age and older. *Neurology* 100 e1691–e1701. 10.1212/WNL.0000000000207077 36849448 PMC10115503

[B6] Campabadal A. Uribe C. Segura B. Baggio H. C. Abos A. Garcia-Diaz A. I. (2017). Brain correlates of progressive olfactory loss in Parkinson’s disease. *Parkinsonism Relat. Disord.* 41 44–50. 10.1016/j.parkreldis.2017.05.005 28522171

[B7] Domellöf M. E. Lundin K. F. Edström M. Forsgren L. (2017). Olfactory dysfunction and dementia in newly diagnosed patients with Parkinson’s disease. *Parkinsonism Relat. Disord.* 38 41–47. 10.1016/j.parkreldis.2017.02.017 28242255

[B8] Doty R. L. (2019). Psychophysical testing of smell and taste function. *Handb. Clin. Neurol.* 164 229–246. 10.1016/B978-0-444-63855-7.00015-0 31604550

[B9] Doty R. L. Deems D. A. Stellar S. (1988). Olfactory dysfunction in parkinsonism: A general deficit unrelated to neurologic signs, disease stage, or disease duration. *Neurology* 38 1237–1244. 10.1212/wnl.38.8.1237 3399075

[B10] Doty R. L. Shaman P. Dann M. (1984). Development of the University of Pennsylvania smell identification test: A standardized microencapsulated test of olfactory function. *Physiol. Behav.* 32 489–502. 10.1016/0031-9384(84)90269-5 6463130

[B11] Ercoli T. Masala C. Cadeddu G. Mascia M. M. Orofino G. Gigante A. F. (2022). Does olfactory dysfunction correlate with disease progression in Parkinson’s Disease? A systematic review of the current literature. *Brain Sci.* 12:513. 10.3390/brainsci12050513 35624900 PMC9139278

[B12] Fujio H. Inokuchi G. Kuroki S. Tatehara S. Katsunuma S. Kowa H. (2020). Three-year prospective study on olfaction of patients with Parkinson’s disease. *Auris Nasus Larynx* 47 899–904. 10.1016/j.anl.2019.08.008 31506174

[B13] Fullard M. E. Morley J. F. Duda J. E. (2017). Olfactory dysfunction as an early biomarker in Parkinson’s Disease. *Neurosci. Bull.* 33 515–525. 10.1007/s12264-017-0170-x 28831680 PMC5636737

[B14] Fullard M. E. Tran B. Xie S. X. Toledo J. B. Scordia C. Linder C. (2016). Olfactory impairment predicts cognitive decline in early Parkinson’s disease. *Parkinsonism Relat. Disord.* 25 45–51. 26923521 10.1016/j.parkreldis.2016.02.013 PMC4825674

[B15] Goetz C. G. Tilley B. C. Shaftman S. R. Stebbins G. T. Fahn S. Martinez-Martin P. (2008). Movement Disorder Society-sponsored revision of the Unified Parkinson’s Disease Rating Scale (MDS-UPDRS): Scale presentation and clinimetric testing results. *Mov. Disord.* 23 2129–2170. 19025984 10.1002/mds.22340

[B16] He R. Zhao Y. He Y. Zhou Y. Yang J. Zhou X. (2020). Olfactory dysfunction predicts disease progression in Parkinson’s Disease: A longitudinal study. *Front. Neurosci.* 14:569777. 10.3389/fnins.2020.569777 33381006 PMC7768001

[B17] Herting B. Schulze S. Reichmann H. Haehner A. Hummel T. (2008). A longitudinal study of olfactory function in patients with idiopathic Parkinson’s disease. *J. Neurol.* 255 367–370. 10.1007/s00415-008-0665-5 18343969

[B18] Horsager J. Andersen K. B. Knudsen K. Skjærbæk C. Fedorova T. D. Okkels N. (2020). Brain-first versus body-first Parkinson’s disease: A multimodal imaging case-control study. *Brain* 143 3077–3088. 10.1093/brain/awaa238 32830221

[B19] Horsager J. Knudsen K. Sommerauer M. (2022). Clinical and imaging evidence of brain-first and body-first Parkinson’s disease. *Neurobiol. Dis.* 164:105626. 10.1016/j.nbd.2022.105626 35031485

[B20] Iranzo A. Marrero-González P. Serradell M. Gaig C. Santamaria J. Vilaseca I. (2021). Significance of hyposmia in isolated REM sleep behavior disorder. *J. Neurol.* 268 963–966. 32968939 10.1007/s00415-020-10229-3

[B21] Jellinger K. A. (2019). Is Braak staging valid for all types of Parkinson’s disease? *J. Neural Transm.* 126 423–431. 29943229 10.1007/s00702-018-1898-9

[B22] Jiang R.-S. Su M.-C. Liang K.-L. Shiao J.-Y. Wu S.-H. Hsin C.-H. (2010). A pilot study of a traditional Chinese version of the University of pennsylvania smell identification test for application in Taiwan. *Am. J. Rhinol. Allergy* 24 45–50. 10.2500/ajra.2010.24.3388 20109324

[B23] Lee D. H. Oh J. S. Ham J. H. Lee J. J. Lee I. Lee P. H. (2015). Is normosmic Parkinson disease a unique clinical phenotype? *Neurology* 85 1270–1275. 26354986 10.1212/WNL.0000000000001999

[B24] Lewis M. M. Harkins E. Lee E. Y. Stetter C. Snyder B. Corson T. (2020). Clinical Progression of Parkinson’s Disease: Insights from the NINDS common data elements. *J. Parkinsons Dis.* 10 1075–1085. 10.3233/JPD-201932 32538866 PMC8177750

[B25] Lin C. Y. Chang T. Y. Chang M. H. (2022). Dysosmia is a predictor of motor function and quality of life in patients with Parkinson’s Disease. *J. Pers. Med.* 12 754. 10.3390/jpm12050754 PMC9143120 35629176

[B26] Ma H. I. Hwang W. J. Chen-Sea M. J. (2005). Reliability and validity testing of a Chinese-translated version of the 39-item Parkinson’s Disease Questionnaire (PDQ-39). *Qual. Life Res.* 14 565–569. 10.1007/s11136-004-0687-0 15892447

[B27] Martinez-Martin P. Rodriguez-Blazquez C. Kurtis M. M. Chaudhuri K. R. (2011). The impact of non-motor symptoms on health-related quality of life of patients with Parkinson’s disease. *Mov. Disord.* 26 399–406. 21264941 10.1002/mds.23462

[B28] Masala C. Solla P. Liscia A. Defazio G. Saba L. Cannas A. (2018). Correlation among olfactory function, motors’ symptoms, cognitive impairment, apathy, and fatigue in patients with Parkinson’s disease. *J. Neurol.* 265 1764– 1771. 29804147 10.1007/s00415-018-8913-9

[B29] Meusel T. Westermann B. Fuhr P. Hummel T. Welge-Lüssen A. (2010). The course of olfactory deficits in patients with Parkinson’s disease–a study based on psychophysical and electrophysiological measures. *Neurosci. Lett.* 486 166–170. 10.1016/j.neulet.2010.09.044 20858529

[B30] Mundiñano I. C. Caballero M. C. Ordóñez C. Hernandez M. Dicaudo C. Marcilla I. (2011). Increased dopaminergic cells and protein aggregates in the olfactory bulb of patients with neurodegenerative disorders. *Acta Neuropathol.* 122 61–74. 21553300 10.1007/s00401-011-0830-2

[B31] Nasreddine Z. S. Phillips N. A. Bédirian V. Charbonneau S. Whitehead V. Collin I. (2005). The montreal cognitive assessment, MoCA: A brief screening tool for mild cognitive impairment. *J. Am. Geriatr. Soc.* 53 695–699. 10.1111/j.1532-5415.2005.53221.x 15817019

[B32] Pasquini J. Brooks D. J. Pavese N. (2021). The cholinergic brain in Parkinson’s Disease. *Mov. Disord. Clin. Pract.* 8 1012–1026. 34631936 10.1002/mdc3.13319 PMC8485627

[B33] Peto V. Jenkinson C. Fitzpatrick R. Greenhall R. (1995). The development and validation of a short measure of functioning and well being for individuals with Parkinson’s disease. *Qual. Life Res.* 4 241–248. 10.1007/BF02260863 7613534

[B34] Picillo M. Pellecchia M. T. Erro R. Amboni M. Vitale C. Iavarone A. (2014). The use of University of Pennsylvania smell identification test in the diagnosis of Parkinson’s disease in Italy. *Neurol Sci.* 35 379–383. 23975523 10.1007/s10072-013-1522-6

[B35] Postuma R. B. Berg D. Stern M. Poewe W. Olanow C. W. Oertel W. (2015). MDS clinical diagnostic criteria for Parkinson’s disease. *Mov. Disord.* 30 1591–1601. 26474316 10.1002/mds.26424

[B36] Solla P. Masala C. Ercoli T. Frau C. Bagella C. Pinna I. (2023). Olfactory impairment correlates with executive functions disorders and other specific cognitive dysfunctions in Parkinson’s Disease. *Biology* 12:112. 10.3390/biology12010112 PMC9855400 36671804

[B37] Tsuboi Y. Wszolek Z. K. Graff-Radford N. R. Cookson N. Dickson D. W. (2003). Tau pathology in the olfactory bulb correlates with Braak stage, Lewy body pathology and apolipoprotein epsilon4. *Neuropathol. Appl. Neurobiol.* 29 503–510. 10.1046/j.1365-2990.2003.00453.x 14507342

[B38] Wang R. Lian T. He M. Guo P. Yu S. Zuo L. (2023). Clinical features and neurobiochemical mechanisms of olfactory dysfunction in patients with Parkinson disease. *J. Neurol.* [Online ahead of print]. 10.1007/s00415-023-12122-1 38151574

[B39] Yu C. Y. Wu R. M. (2014). Application of the University Of Pennsylvania smell identification test (traditional Chinese version) for detecting olfactory deficits in early Parkinson’s disease in a Taiwanese cohort. *J. Parkinsons Dis.* 4 175–180. 10.3233/JPD-130309 24796234

