# Supplementary material for: Impact of olfactory function on the trajectory of cognition, motor function, and quality of life in Parkinson’s disease
Source: Front Aging Neurosci. 2024 Mar 4;16:1329551. doi: 10.3389/fnagi.2024.1329551 (PMC10944858; doi:10.3389/fnagi.2024.1329551)
Supplement: Supplementary file 1 [file Data_Sheet_1.PDF]

Supplementary Material

1 Supplementary Data

1.1 Supplementary Tables

Table S1. Adjusted olfactory's impact on clinical outcomes over time: LMM analysis

	MoCA		Part III	
	B	*p*	*B*	*p*
Total				
UPSIT	0.1728	0.0344*	-0.4152	0.0380*
Age	-0.1401	0.0223*	0.2885	0.0501
Sex	2.1231	0.0758	-0.4144	0.8842
Duration	-0.0097	0.2951	0.0029	0.8971
Anosmia				
UPSIT	0.3191	0.0724	0.1691	0.6710
Age	-0.1037	0.3073	0.2845	0.2180
Sex	3.1137	0.1036	-3.8800	0.3610
Duration	-0.0089	0.4719	-0.0163	0.5570
Non-anosmia				
UPSIT	-0.0977	0.3422	-0.6653	0.0352*
Age	-0.2019	0.0046*	0.2897	0.1573
Sex	0.8947	0.5145	2.1916	0.6013
Duration	-0.0090	0.5351	0.0356	0.4232

The model was adjusted for age, sex and duration, with "duration" denoting time since symptom onset. Anosmia: baseline UPSIT < 19; Non-anosmia: baseline UPSIT ≥ 19.

B, beta coefficient; LMM, linear mixed effect model; MoCA, Montreal Cognitive Assessment; UPSIT, traditional Chinese version of the University of Pennsylvania Smell Identification Test. * $p < 0.05$

Table S2. Anosmia vs. non-anosmia: initial comparison based on first visit and aggregate of the two visits with 2 completed assessments

	Visit 1			Visit 2	
	Anosmia n = 25	Non-anosmia n = 19	*p*	Anosmia n = 25	Non-anosmia n = 19
Age, year	63 (60–70)	70 (58–75)	0.455	65 (60–71)	65 (59–77)
Sex, male (%)	18 (72%)	11 (58%)	0.357	18 (72%)	11 (58%)
Sex, female (%)	7 (28%)	8 (42%)		7 (28%)	8 (42%)
Duration, month	55 (36–117)	31 (12–56)	0.017*	79 (58–131)	52 (29–71)
Follow-up, month	0 (0–0)	0 (0–0)	1.000	16 (14–21)	15 (14–22)
LEDD, mg	713(168–1076)	300(100–450)	0.007*	798(423–1297)	400(250–639)
UPSIT	15 (12–17)	21 (20–25)	0.000*		
MOCA	27 (22–29)	27 (24–29)	0.933	24 (20–28)	27 (23–29)
M-UPDRS					
Total	64 (39–82)	45 (28–57)	0.027*	50 (37–82)	47 (34–53)
Part III	34 (22–46)	25 (18–36)	0.081	31 (23–42)	29 (23–36)
PDQ-39					
SI	22 (11–35)	22 (6–29)	0.292	14 (6–33)	14 (9–27)
ADL	17 (6–38)	4 (0–21)	0.056	13 (0–27)	4 (0–21)
COG	25 (19–50)	19 (13–44)	0.273	19 (6–47)	19 (0–38)

Data are presented as median (Q1–Q3), with "duration" denoting time since symptom onset and "follow-up" representing time from initial visit. Anosmia: baseline UPSIT < 19; Non-anosmia: baseline UPSIT ≥ 19.

ADL, activities of daily living; COG, cognitions; LEDD, Levodopa equivalent daily dose; MoCA, Montreal Cognitive Assessment; M-UPDRS, Movement Disorder Society-sponsored revision of the Unified Parkinson's Disease Rating Scale; PDQ-39, Chinese-translated version of 39-item Parkinson's Disease Questionnaire; SI, summary index; UPSIT, traditional Chinese version of the University of Pennsylvania Smell Identification Test.
* $p < 0.05$

Table S3. Clinical assessment trajectories in the two groups over the first two visits: Wilcoxon signed-rank analysis

	Anosmia visit 2 vs. visit 1		Non-anosmia visit2 vs. visit 1	
	median Δ	*p*	median Δ	*p*
LEDD	180	<0.001*	150	<0.001*
UPSIT	-1.5	0.062	-1.5	0.283
MoCA	-2.5	0.003*	-0.5	0.449
M-UPDRS				
Total	-3.5	0.429	0.5	0.862
Part III	-1.0	0.667	1.0	0.585
PDQ-39				
SI	-3.4	0.059	-0.7	0.794
ADL	-6.3	0.175	-9.4	0.284
COG	-2.1	0.129	0.0	0.638

The anosmia group displayed a decline in MoCA score from baseline (median Δ = -2.5, $p = 0.003$). Anosmia: baseline UPSIT < 19; Non-anosmia: baseline UPSIT ≥ 19.

ADL, activities of daily living; COG, cognitions; LEDD, Levodopa equivalent daily dose; median Δ, Hodges-Lehmann median difference; MoCA, Montreal Cognitive Assessment; M-UPDRS, Movement Disorder Society-sponsored revision of the Unified Parkinson's Disease Rating Scale; PDQ-39, Chinese-translated version of 39-item Parkinson's Disease Questionnaire; SI, summary index; UPSIT, traditional Chinese version of the University of Pennsylvania Smell Identification Test. * $p < 0.05$

Table S4. Olfaction and time interaction effects on clinical assessment trajectories of the first two visits: GEE analysis

	Crude model				Adjusted model			
	olfaction		olfaction × time		olfaction		olfaction × time	
	crude *B*	*p*	crude *B*	*p*	adjusted *B*	*p*	adjusted *B*	*p*
Age	-1.6	0.593	0.0	0.831				
Duration	37.9	0.025*	0.0	0.976				
LEDD	346.2	0.012*	29.2	0.683				
MOCA	2.0	0.240	-2.4	0.051	2.3	0.142	-2.3	0.062
UPSIT	-8.4	<0.001*	-0.3	0.838	-8.1	<0.001*	-0.2	0.868
M-UPDRS								
Total	22.3	0.051	-4.2	0.510	18.1	0.129	-4.6	0.492
Part III	12.1	0.063	-3.9	0.241	12.6	0.052	-4.0	0.236
PDQ-39								
SI	9.2	0.299	-3.7	0.440	4.6	0.607	-4.0	0.432
ADL	14.1	0.233	-4.2	0.483	8.1	0.509	-4.7	0.476
COG	2.8	0.828	1.4	0.852	-4.1	0.747	1.0	0.897

The olfaction effects were compared between anosmia and non-anosmia groups. Adjustments were made for age, sex, disease duration, and LEDD. Data indicates a faster cognitive decline in the anosmia group (olfaction × time effect on MoCA scores adjusted $B = -2.3$, $p = 0.062$). Anosmia: baseline UPSIT < 19; Non-anosmia: baseline UPSIT ≥ 19.

ADL, activities of daily living; *B*, beta coefficient; COG, cognitions; GEE, Generalized estimating equation; LEDD, Levodopa equivalent daily dose; MoCA, Montreal Cognitive Assessment; M-UPDRS, Movement Disorder Society-sponsored revision of the Unified Parkinson’s Disease Rating Scale; PDQ-39, Chinese-translated version of 39-item Parkinson’s Disease Questionnaire; SI, summary index; UPSIT, traditional Chinese version of the University of Pennsylvania Smell Identification Test. * $p < 0.05$

Table S5. Longitudinal correlation of clinical assessments with demographic factors and UPSIT over the first two visits: Rmcorr analysis

	MoCA		Part III		COG		ADL	
	r_{rm}	p	r_{rm}	p	r_{rm}	p	r_{rm}	p
Anosmia								
Age	-0.517	0.007*	0.018	0.932	-0.306	0.129	-0.219	0.282
Duration	-0.477	0.014*	-0.066	0.750	-0.341	0.089	-0.322	0.109
LEDD	-0.165	0.422	-0.442	0.024*	-0.397	0.045*	-0.643	<0.001*
UPSIT	0.034	0.867	0.134	0.514	0.261	0.198	0.394	0.047*
Non-anosmia								
Age	-0.295	0.207	0.375	0.103	-0.167	0.481	-0.009	0.971
Duration	-0.249	0.290	0.326	0.161	-0.178	0.454	-0.079	0.742
LEDD	-0.008	0.973	0.023	0.925	-0.147	0.537	-0.330	0.156
UPSIT	-0.077	0.748	-0.441	0.051	-0.341	0.142	-0.209	0.376

Table S4 shows a negative correlation between UPSIT scores and part III scores in the non-anosmia group (r_{rm} = -0.441, p = 0.051).
Anosmia: baseline UPSIT < 19; Non-anosmia: baseline UPSIT ≥ 19.

ADL, activities of daily living of PDQ-39; COG, cognitions of PDQ-39; LEDD, Levodopa equivalent daily dose; MoCA, Montreal Cognitive Assessment; Part III, Part III of Movement Disorder Society-sponsored revision of the Unified Parkinson's Disease Rating Scale; PDQ-39, Chinese-translated version of 39-item Parkinson's Disease Questionnaire; Rmcorr, repeated measures correlation; r_{rm}, coefficient or repeat measurement correlation; UPSIT, traditional Chinese version of the University of Pennsylvania Smell Identification Test. * $p < 0.05$

Supplementary Figures

Fig. S1

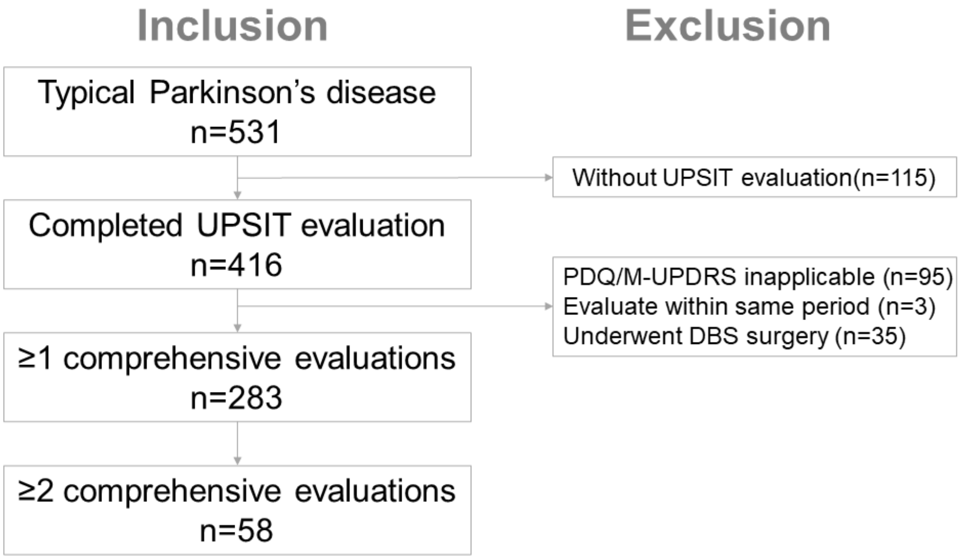

The Fig. S1 indicate flow diagram of inclusion in this study

DBS, deep brain stimulation; M-UPDRS, Movement Disorder Society-sponsored revision of the Unified Parkinson's Disease Rating Scale; PDQ-39, Chinese-translated version of 39-item Parkinson's Disease Questionnaire; UPSIT, traditional Chinese version of the University of Pennsylvania Smell Identification Test.
